# Predictors of Serotonin Syndrome in Acute Poisoning with 5-Hydroxytryptamine Modulators

**DOI:** 10.3390/toxics12080550

**Published:** 2024-07-30

**Authors:** Asmaa F. Sharif, Mubarak Nasir M. Almulhim, Hadi Mohamed A. Almosabeh, Mohammed Essam A. Alshammasy, Ali Mohammed A. Aljeshi, Taher Mohammed A. Mufti, Shahd AlNasser, Khalid A. Al-Mulhim, Yousef A. AlMubarak

**Affiliations:** 1 Department of Clinical Medical Sciences, College of Medicine, Dar AL-Uloom University, Al Falah, Riyadh 13314, Saudi Arabia; 2 Forensic Medicine and Clinical Toxicology Department, Faculty of Medicine, Tanta University, El Bahr St., Tanta 31111, Egypt; 3 College of Medicine, Dar AL-Uloom University, Al Falah, Riyadh 13314, Saudi Arabia; brooknasser@gmail.com (M.N.M.A.); hadi.2220@hotmail.com (H.M.A.A.); m7ammedes03@gmail.com (M.E.A.A.); alimaljeshi@gmail.com (A.M.A.A.); drtahermufti@gmail.com (T.M.A.M.); 4 Saudi Food and Drug Authority, Hittin, Riyadh 13513, Saudi Arabia; snnasser@sfda.gov.sa; 5 Emergency Medicine Department, King Fahad Medical City, Sulimaniyah, Riyadh 12231, Saudi Arabia; kalmulhim@kfmc.med.sa (K.A.A.-M.); yalmubarak@kfmc.med.sa (Y.A.A.)

**Keywords:** serotonin syndrome, selective serotonin re-uptake inhibitors, 5-hydroxytryptophan, Hunter Serotonin Toxicity Criteria, serotonergic drugs, serotonin toxicity

## Abstract

5-Hydroxytryptamine (5-HT) modulators are commonly prescribed medications with potentially life-threatening outcomes, particularly serotonin syndrome (SS). Early prediction of SS is critical not only to avoid lethal drug combinations but also to initiate appropriate treatment. The present work aimed to recognize the significant predictors of SS through a retrospective cross-sectional study that was conducted among patients exposed to an overdose of 5-HT modulators and admitted to a poison control center where 112 patients were enrolled. Of them, 21 patients were diagnosed with SS, and 66.7% of patients with SS were exposed to long-term co-ingestion. There was a noticeable surge in SS between April and May, and 52.4% of patients who suffered from SS were admitted after suicidal exposure (*p* < 0.05). Patients with SS showed severe presentation indicated by high-grade poison severity scores (PSS) and low Glasgow coma scales (GCS). PSS was a significant predictor of SS with an area under the curve of 0.879. PCO_2_, pulse, GCS, HCO_3_, and erythrocytic count were other significant predictors of SS. Combinations of serotonergic agents increase the likelihood of developing SS. Clinicians should be vigilant when prescribing a combination of serotonergic therapy, particularly for patients on illicit sympathomimetic and over-the-counter medications like dextromethorphan.

## 1. Introduction

Serotonin, also known as 5-hydroxytryptamine (5-HT), is a chemical neurotransmitter present in both the central and peripheral nervous systems. It was initially discovered in the 1940s. Serotonin has a significant impact on various physiological functions in humans. These functions include the regulation of mood, sleep–wake cycle, appetite suppression, memory, emesis, breathing, cognition, blood coagulation, libido, and others [1].

Serotonin modulators modify the serotonin pathway primarily in the neurons located within the midbrain, pons, and medulla [2]. Serotonin modulators are commonly prescribed medications for diverse disorders, including depression, anxiety disorders, schizophrenia, chronic pain, fibromyalgia, sleep disorders, and eating disorders [3,4,5]. In addition, some serotonin modulators are among the illicit drugs abused [6].

The diverse uses of 5-HT modulators make them readily available for potential misuse or overdose. While most patients benefit from these drugs when taken as prescribed, acute poisoning events are occurring with increasing frequency. The clinical significance lies in the potential for severe outcomes and potentially life-threatening symptoms, including seizures and cardiovascular complications, necessitating prompt medical intervention [6].

Serotonin toxicity is the most severe life-threatening form of serotonin modulator toxicity. Serotonin syndrome (SS) is characterized by agitation, tachycardia, rigidity, seizures, hyperthermia, rhabdomyolysis, dysrhythmias, and death [7]. Aside from SS, which is associated with increased serotonin levels in the brain, the toxicity of serotonin modulators encompasses other severe manifestations. Mental and behavioral changes, automimic instability, neuromuscular disability, and cardiotoxicity are well-recognized toxic manifestations [8].

There are several challenges in the diagnosis and management of SS. The true incidence of SS is undetermined. This syndrome is commonly underdiagnosed or overlooked by physicians, owing to their unfamiliarity with this condition. Moreover, the self-limiting nature of SS limits its tackling in randomized clinical control trials [9]. SS is a clinical diagnosis that depends on history of exposure and clinical findings. No specific biomarker exists to confirm the diagnosis, and laboratory tests are used to rule out other diseases [10].

Lack of standardized definitions and inconclusive diagnostic criteria of SS have been other reported challenges [11]. Although the Hunter Serotonin Toxicity Criteria (HSTC) Decision Rules were privileged for being more precise, these criteria are inapplicable to mild cases of SS, which are difficult to distinguish from numerous other medical conditions and side effects [10]. Other similar medical conditions include malignant hyperthermia, neuroleptic malignant syndrome, anticholinergic toxicity, sympathomimetic poisoning, meningitis, and encephalitis [12]. Another challenge in the management of SS is the lack of specific antidotes. In severe cases, substances with 5-HT_2A_ antagonistic activity such as cyproheptadine or chlorpromazine are recommended [13].

Early prediction of SS is critical not only to avoid lethal drug combinations but also to initiate appropriate treatment, as symptoms of SS progress quickly [14]. Considering the paucity of studies on symptomatology, diagnosis, and complications of SS, especially in the context of acute drug poisoning [6,7], the current study aimed to define the epidemiological profile, clinical features, and risk factors associated with SS among patients exposed to drug overdose with serotonin modulators and, moreover, to identify the significant predictors of SS among this category of patients.

## 2. Materials and Methods

### 2.1. Study Design and Setting 

The current study is a retrospective cross-sectional study that was conducted among adult patients diagnosed with acute drug poisoning and admitted to King Fahad Medical City (KFMC) Emergency Department between January 2020 and December 2022.

### 2.2. Sampling and Sample Size Calculations

Convenience sampling was adopted to approach all available medical records of patients fulfilling the inclusion criteria. However, to ensure that the studied sample was sufficient to answer the research question, the sample size was calculated using Open Epi software Version 3, open-source calculator-SSPropor (https://www.openepi.com/, accessed on 24 July 2024). The true incidence of SS is challenging to assess owing to the lack of awareness of this syndrome among physicians. It was reported that about 85% of physicians are unaware of this syndrome [15]. Prevalences ranged from very low values (0.09–0.005) [16] to higher values (14–16%) [17]. However, to ensure that the studied sample was sufficient to investigate the outcome, we calculated the sample size based on a midway prevalence of SS (7.8%) reported by Prakash et al. in their recent study diagnosing SS using the HSTC Decision Rules among patients admitted to an intensive care unit (ICU) [18]. The minimum sample size was estimated to be at least 111 patients; we obtained 112 patients.

### 2.3. Inclusion Criteria

From the database of KFMC, all the adult patients diagnosed with acute poisoning with a single serotonin modulator who presented and were admitted during the stated period were obtained. We searched nine categories of serotonin modulators as per Sun-edelstein et al., including selective serotonin re-uptake inhibitors (SSRIs), serotonin–norepinephrine re-uptake inhibitors (SNRIs), tricyclic antidepressants (TCA), monoamine oxidase inhibitors (MAOIs), opioids, serotonin releasers, serotonin precursors, triptans, and miscellaneous drugs including antihistamines and lithium [14]. All patients exposed acutely to single-type serotonin modulators with complete medical records were considered eligible regardless of the manner or circumstances of exposure. The diagnosis of SS was confirmed based on HSTC Decision Rules [11]. 

### 2.4. Exclusion Criteria 

Among the exclusion criteria were recent infection or substance withdrawal and being on 5-HT antagonists, including those on neuroleptic agents [19]. Patients who suffered from co-morbid cardiac or neurological disorders were excluded, in addition to the patients with unconfirmed diagnoses and missing or incomplete medical records. 

### 2.5. Compliance with Ethical Standards

The current study was conducted after obtaining Institutional Review Board (IRB) approval from KFMC (IRB Log Number: 23-615). According to the Declaration of Helsinki and its later amendment, which states that the interest of privacy and safety to the patient is over the interests of science and society, medical records were handled anonymously, and patient confidentiality was preserved using a coding system for case report forms. IRB exempted the informed consent due to the observational nature of the study.

### 2.6. Data Collection Tool

The patients enrolled in the current study were classified into two groups: a group with no SS and a group of SS diagnosed by the HSTC Decision Rules [11]. For every included patient, a predesigned case report form was completed by two independent investigators, including the demographic data like age and sex, the exposure history involving the manner of exposure, the name and group of the used drug, and the delay time from exposure until receiving the emergency treatment. 

Vital signs and patient scoring, including the Glasgow Coma Scale (GCS) and Poison Severity Score (PSS), were reported for every patient on admission. However, the presenting complaints and the clinical findings noticed during the examination were reported. Furthermore, we reported the results of performed investigations including random blood glucose level, serum electrolytes, total bilirubin level, liver transaminases (SGOT, SGPT), serum urea and creatinine levels, arterial blood gas analysis, and complete blood count (CBC). Therapeutic regimens were reported, including treatment with 5-HT_2A_ antagonists, mechanical ventilation, or need for ICU admission. The length of hospital stay from admission until discharge was reported.

### 2.7. Data Analysis

The collected data were organized and analyzed using statistical package for the social sciences (SPSS) for Windows, version 28 (IBM Corp., Armonk, NY, USA). The Shapiro–Wilk normality test was performed to assess the distribution of the numerical data. Quantitative data were represented by mean, standard deviation (SD), range, median, and interquartile ranges (25th–75th percentiles). Qualitative data were presented by number and percent. The results were tabulated, grouped, and statistically analyzed: Independent *t* Test (t) for comparing parametric quantitative variables and Mann–Whitney U Test (U) for comparing nonparametric quantitative variables between the two groups. Pearson Chi-square test (χ^2^) was used to detect whether there was a significant association between different categorical variables. It was replaced by the Fischer exact or Monte Carlo exact test when it was inappropriate. 

Univariate and multivariate regression analyses were performed to study the relation between several variables as a predictor for SS. Initially, we conducted univariate analyses to identify potential predictors of serotonin syndrome (SS) from various clinical and biochemical parameters showing significance in the baseline analyses. Predictors that were significant in univariate analysis (*p* < 0.05) were subsequently included in a multivariate logistic regression model. To avoid co-linearity the PSS was assessed as a separate predictor using univariate analysis. 

Receiver operating characteristic (ROC) curve was used to measure the areas under the curve (AUC) and the accuracy, sensitivity, and specificity of the different predictors. Positive predictive value (PPV) and negative predictive value (NPV) were calculated. *p* < 0.05 and confidence interval of 95% were considered significant. To establish the reliability of the identified predictors, sensitivity analysis was conducted to assess the robustness of the findings to changes in model assumptions. This was conducted through applying an alternative statistical model in the form of binary logistic regression to the dataset, to assess the significance of key predictors previously identified by the original model.

## 3. Results

The current study was conducted enrolling 112 patients. Of them, 21 patients were diagnosed with SS using HSTC, representing 18.8% of the studied patients. Table 1 demonstrates the sensitivities and specificities of diagnosing SS by a clinical toxicologist and the decision to administer 5-HT_2A_ antagonists compared to diagnosis through HSTC, which is the golden standard tool for diagnosing SS. While the diagnosis of SS by the clinician showed perfect specificity, depending on clinical sense alone can inappropriately exclude true patients suffering from SS (sensitivity of 76.2%). On the other hand, diagnosing the SS based on the treatment with a 5-HT_2A_ antagonist yielded less sensitive and specific results with a high probability of misdiagnosis or overdiagnosis.

**Figure 1:**
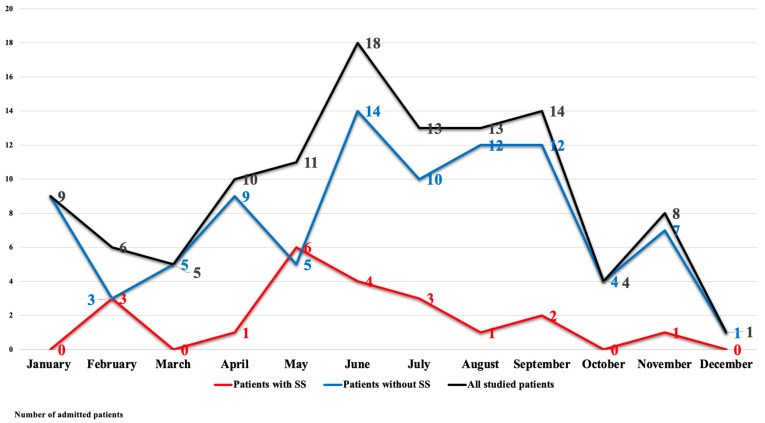
Distribution of admitted patients according to the month of admission.

Figure 1 shows significant variations in the month of admission between the patients who suffered from SS and those who did not (*p* = 0.041). There was a noticeable surge in SS between April and May where the latter represented the peak of SS (n = 6, 28.6%). The mean age of studied patients was significantly higher among patients with SS. More than half of patients who suffered from SS (52.4%) were admitted as a result of suicidal exposure, and about 28.6% were admitted following exposure to obtain inebriation (*p* = 0.004). SS was significantly associated with long-term co-ingestion, in which 66.7% of patients diagnosed with SS were on other agents. SS was also associated with significantly higher blood pressure measurements, temperature, respiratory rates, and pulse. Furthermore, patients diagnosed with SS showed more severe presentations indicated by lower GCS and higher grades of PSS (*p* < 0.001), as shown in Table 2.

**Figure 2:**
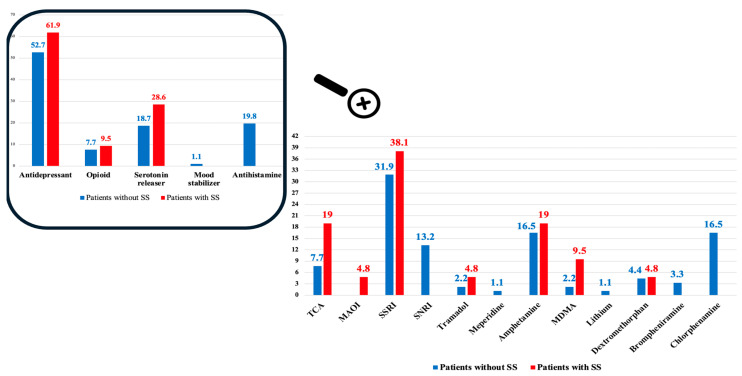
Proportion of admitted patients according to the type of acute drug poisoning.

Regarding the acute poisoning, Figure 2 depicts that five categories of serotonin modulators were identified, including antidepressants, opioids, serotonin releasers, mood stabilizers, and antihistamines. The antidepressants represented the most commonly reported agent in both groups, reported in 61 patients (54.5%) of the studied patients, while the mood stabilizers were the least frequent agent reported in one patient. SSRIs represented the most frequently consumed drugs on an acute basis (31.9% without SS and 38.1% of patients with SS), followed by amphetamines in 17% (16.5% without SS and 19% of patients with SS).

**Figure 3:**
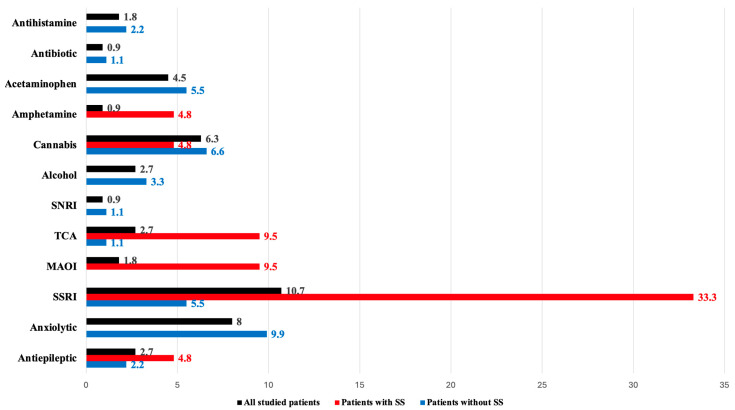
Proportion of long-term co-ingested agents reported by the studied patients.

**Figure 4:**
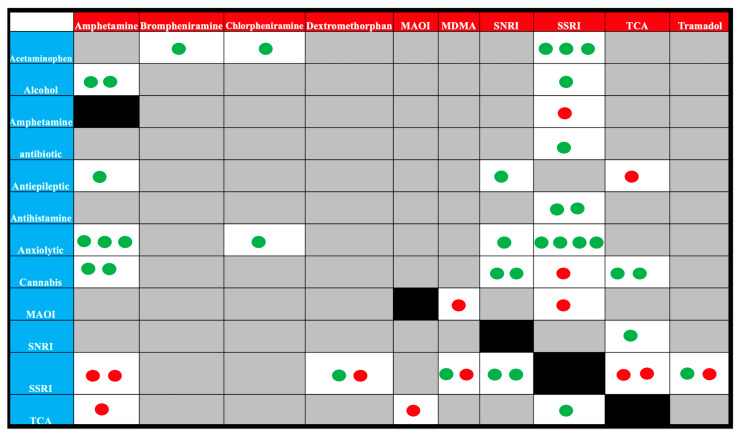
Drug combinations among 49 patients diagnosed with acute poisoning with 5-HT modulators. The red boxes represent the drugs consumed acutely, while the blue boxes represent long-term therapies. The number of circles reflects the number of drug combinations encountered. The red circle refers to a drug–drug combination associated with serotonin syndrome, while the green circle refers to a drug–drug combination not associated with serotonin syndrome. Gray cells mean the intersected agent’s combination was not encountered. Black cells refer to inability to assess DDIs as the acutely consumed and long-term agents are the same. For example, two patients exposed to an overdose of dextromethorphan were on SSRIs. One of these patients suffered from SS, while the other patient did not. Also, three patients on anxiolytics were exposed to an overdose of amphetamine. None of them suffered from SS.

Regarding the long-term used drugs, about 43.8% of patients (n = 49) were exposed to additive agents apart from serotonin modulators. Figure 3 shows that twelve long-term drug groups were recognized. About 33% of the studied patients were already on SSRIs, representing 10.7% of the studied patients, followed by anxiolytics at 8%, cannabis at 6.3%, and acetaminophen at 4.5%. As Figure 4 demonstrates the drug combinations were reported in 49 patients. SS was a significant finding among patients on long-term therapy with MAOIs, amphetamines, TCAs, and SSRIs (*p* < 0.001). On the other side, anxiolytics, cannabinoids, acetaminophen, alcohol, antihistamines, and antibiotics were relatively safe drugs. Nevertheless, in the context of co-ingestion, acute exposure to MAOIs, MDMA, TCA, tramadol, and dextromethorphan was more frequent in patients with SS compared to SNRIs. The most dangerous combinations were amphetamine with SSRIs or TCAs, dextromethorphan and SSRI, MAOI with TCA, MDAM and SSRI, SSRI with cannabis, TCA and SSRIs, TCA and antiepileptics and combination with tramadol and SSRIs.

Regarding the symptomatology of the studied patients, about 90.5% of patients who suffered from SS presented with GI manifestations and mydriasis. Palpitation, decreased consciousness level, seizures, extrapyramidal manifestations, clonus, diaphoresis, agitation, shivering, tremors, and hyperreflexia were significant findings associated with SS (*p* < 0.05). Additively, only 4.8% of patients with SS did not suffer from cardiac dysrhythmias, as Table 3 reveals. Sinus tachycardia was a significant finding among patients who suffered from SS, which was reported in 95.2% (n = 20) versus 81.7% (n = 17) of the patients without SS (*p* < 0.001). Other forms of dysrhythmias showed comparable presentations among the studied groups.

Table 4 illustrates that patients with SS showed significantly lower bicarbonate and PCO_2_ levels compared to patients without SS (*p* < 0.001). Even though there were significantly higher red blood cell (RBC) counts in patients with SS compared to those without, the RBC count was within the normal in both groups, showing means of 5.1 and 4.7 (million/microliter) for the mentioned groups, respectively. Furthermore, SGPT was significantly higher among patients without SS (*p* = 0.036). As depicted in Table 5, 57.1% of patients with SS received 5-HT_2A_ antagonist compared to 8.8% of patients without SS (*p* < 0.001), summing up to 17.9% of the studied patients (n = 20). All patients of SS were admitted to ICU, and all cases who suffered from respiratory failure and were put on mechanical ventilation suffered from SS (*p* < 0.005). Furthermore, there was a significantly more prolonged hospital stay among patients with SS (mean = 60.6 h) compared to those without (mean = 18.46 h), (*p* < 0.001).

Table 6 shows the clinical characteristics of a single administration and drug combination among the patients diagnosed with SS. Aside from palpitation and chest pain, which were significant findings among patients exposed to single agents, all other clinical features were mutual between the patients exposed to more than one drug (*p* > 0.05). Nevertheless, the patients exposed to drug combinations showed higher means of blood pressure and lower means of GCS. Although a higher proportion of patients exposed to drug combinations exhibited seizures, inducible clonus, shivering, and tremors compared to patients exposed to single agents, these variations did not reach the level of statistical significance. 

Additionally, evaluating the influence of time between exposure and clinical testing on the clinical symptom scores revealed non-significant correlations (*p* > 0.05). Correlating the quantity of delay with GCS yielded coefficients of −0.012 and −0.177 for patients without SS and patients with SS, respectively. Regarding the PSS, we reported similar non-significant coefficients of 0.074 and −0.133 among patients without SS and patients with SS, respectively. This indicates that the timing of the clinical testing, within the range we studied, does not significantly impact the outcomes measured

**Figure 5:**
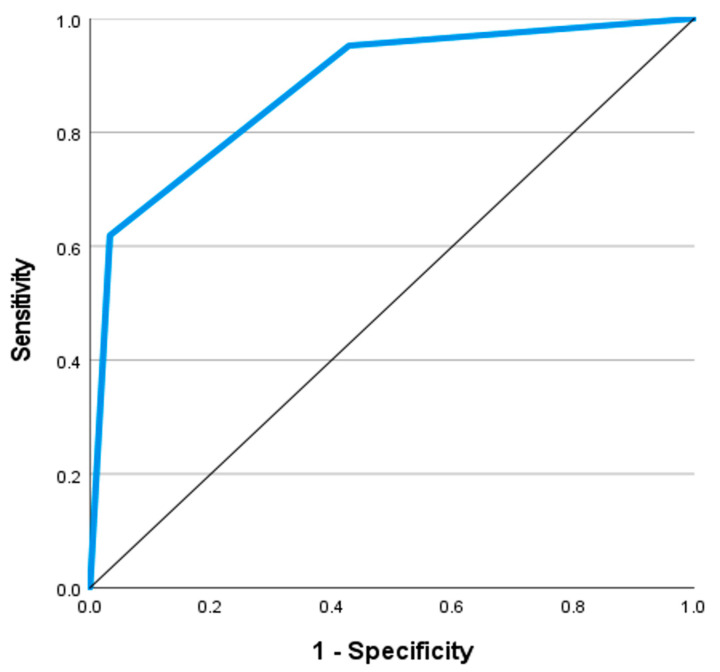
Receiver operating characteristic (ROC) curve analysis for the poison severity score as a predictor of serotonin syndrome.

**Figure 6:**
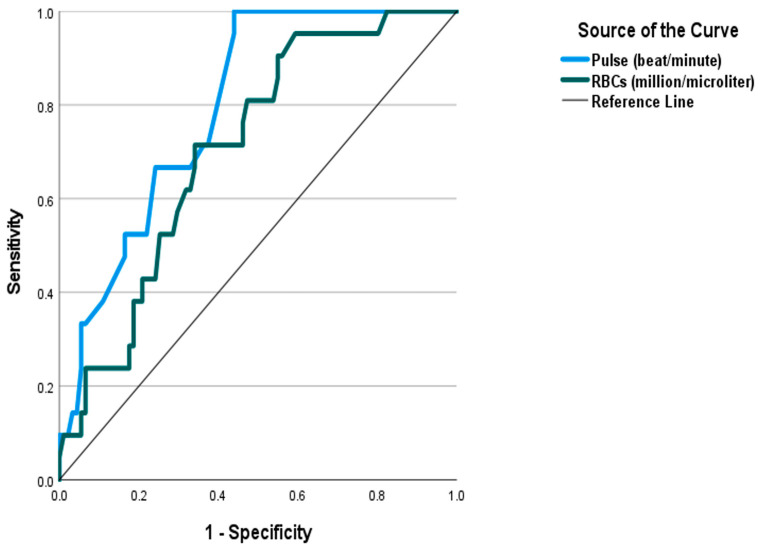
Receiver operating characteristic (ROC) curve analysis for the pulse and red blood cell count as predictors of serotonin syndrome.

**Figure 7:**
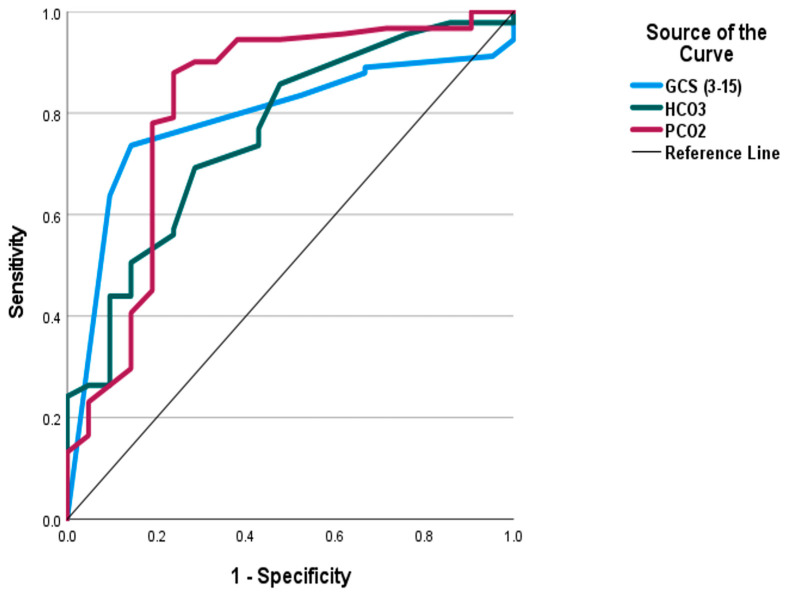
Receiver operating characteristic (ROC) curve analysis for the Glasgow coma scale, HCO_3_, and PCO_2_ as predictors of serotonin syndrome.

The present work conveys that the age, systolic and diastolic blood pressures, respiratory rate, pulse, RBC count, and PSS were significant independent predictors of SS. Increasing the values of each predictor was associated with a higher likelihood of developing SS. On the other side, GCS, HCO_3_, and PCO_2_ showed a paradoxical relationship with SS; they were significant negative predictors. Aside from PSS, which was excluded due to co-linearity, combining the significant predictors indicated that only the pulse, GCS, HCO_3_, PCO_2_, and RBCs remained significant (*p* < 0.05) as Table 7 shows. Table 8 illustrates the ROC curve analysis of the proposed predictors. Figure 5 shows that a PSS of three or more was a significant predictor of SS with an AUC of 0.879, sensitivity of 61.9%, specificity of 96.7%, PPV of 81.3%, and NPV of 91.7%. Figure 6 and Figure 7 demonstrate that PCO_2_ of less than 35.85 was a significant predictor of SS, with an AUC of 0.816 and an accuracy of 78.6%. Pulse of 110 and more, GCS of 13 and less, HCO_3_ less than 21.5 mEq/L, and RBC count > 4.705 (million/microliter) were significant predictors of SS with AUCs ranging between 0.708 and 0.796, and accuracy between 66.9% and 74.1%. Aside from the RBC count, the pairwise comparison of the AUC of PSS, the well-known scoring system, with every single predictor showed no significant variations (*p* > 0.05). Table 9 confirms the reliability of the proposed predictors. Consistent results were observed using alternative statistical model specifications. All predictors showed significant discrimination power (*p* < 0.05), and odds ratio demonstrating that those predictors retained their statistical significance and predictive power under varying assumptions.

## 4. Discussion

The current study aimed to investigate the predictors of SS in 112 patients presented with acute toxicity of 5-HT modulators and to identify the common drugs precipitating this syndrome. With an 18.8% prevalence, it was inferred that SS was more frequent among older patients, suicidal attempters, and those who tried to obtain inebriation. SS was characterized by severe presentation (lower GCS, higher grades PSS) and sympathetic hyperactivity in the form of tachycardia, hypertension, hyperthermia, and tachypnea. Moreover, the PSS was a significant predictor of SS. In addition, low PCO_2_, high pulse, low GCS and HCO_3_, and high RBCs were other significant predictors of SS.

The difficulty in diagnosing SS arises from the lack of standardized diagnostic criteria, the variability of severity in clinical presentation, the lack of knowledge of SS among clinicians [14], and confusion with other disorders like neuroleptic malignant syndrome [19]. In the present work, we used HSTC to diagnose SS, which showed high accuracy. We chose HSTC because of their simplicity and high accuracy compared to other criteria, such as Sternbach’s criteria [11]. Sternbach’s criteria have been criticized for their low specificity and sensitivity, increasing the likelihood that other similar clinical presentations induced by some drugs can be mistaken for SS and overlooking the diagnosis of patients truly suffering from SS. In addition, including ataxia and incoordination seemed to be confusing with cerebellar lesions [14]. 

The present work conveyed that the peak of SS was in May, and there was a noticeable surge in this syndrome between April and May. Though the literature search did not provide any evidence about the association between SS and seasonal changes, there is ample evidence showing seasonal fluctuation in the hypothalamic 5-HT content from a long time ago. Carlsson and colleagues reported that the level of serotonin significantly increases during the spring and summer months. They thought this seasonal fluctuation might explain the variable seasonal prevalence of some affective disorders, suicidal attempts, violent behavior, eating disorders, and alcoholism [20]. These seasonal fluctuations were reported also in healthy people. It was elucidated that the serotonin turnover and availability of serotonin transporter sites are minimal in winter than in summer [21]. Furthermore, Gupta et al. mentioned that the highest level of tryptophan necessary for serotonin synthesis was measured in April and May [21]. 

Serotonin is a neurotransmitter derived from tryptophan. The former is transported into the cells through a unique transport system and is metabolized by MAO-A enzyme to 5-hydroxy indole acetic acid, which is excreted in the urine [11]. Several mechanisms explain SS, including inhibition of serotonin re-uptake, diminution of serotonin metabolism, an increase of serotonin production or neuronal release, and activation of 5-HT_2A_ serotonin receptors [22]. SS results from the concentration-dependent accumulation of serotonin on the central intrasynaptic clefts, contradicting its idiosyncratic predisposition, like the neuroleptic malignant syndrome, which is usually misunderstood [23]. Accumulation of serotonin in a hundred times the average concentration explains the sympathetic storm noticed in these patients [24].

Oates and Sjostrand were the first to describe an illness resulting from excessive serotonin in a patient on MAOI antidepressants who received tryptophan as a sleeping aid [25]. In 1991, Sternbach reviewed literature describing similar cases and established diagnostic criteria for SS [19]. Sternbach criteria were criticized for lacking clinical features that may not have been reported by the reviewed literature, in addition to the appearance of new cases with symptoms or signs not included in the Sternbach criteria [11]. SS comprises a broad spectrum of clinical features rather than a discrete syndrome [26]. Although potentially fatal, no deaths were reported in the studied cohort, which agrees with previous studies stating that the prognosis of SS is almost good unless complicated [14]. 

Alteration of mental status was among the clinical traits described as necessary to diagnose SS. Other features were autonomic instability and neuromuscular changes [27]. However, some patients diagnosed with SS may lack some of these symptoms [14]. Consistent with the current study, hypertension, tachycardia, hyperthermia, mydriasis [11,27], and neuromuscular changes had a statistically significant association with the diagnosis of SS. However, Bodner et al. described orthostatic hypotension as an associated finding [27]. The noticed GI manifestations were attributed to the role of serotonin in regulating gastric motility and smooth muscle tone [14].

There is a tremendous conflicting discrepancy regarding the drugs and drug combinations thought to be associated with SS [11]. In the current study, seven patients were diagnosed with SS after exposure to one drug. However, drug combinations associated with SS were more common, and those exposed to SSRIs, MAOIs, TCAs, and amphetamines were more vulnerable to SS. The concentration-dependent presentation of SS, which was confirmed in animal models, indicates the higher chances of developing SS among drug combination users [28]. Agreeing with the present work, SS occurred following a single overdose [29]. SS due to a combination of MAOIs, known for their drug and food interaction, and SSRIs have been reported repeatedly [30,31]. Bodner et al. described SS as typically occurring following a single exposure to serotonin potentiating agents or their combination with MAOIs [27]. Gillman reported death due to SS after exposure to MAOIs and amphetamines [32]. 

Indeed, although MAOIs are well known for their food and drug interactions, these interactions are more prevalent with old generations of irreversible MAOIs like tranylcypromine, while the reversible inhibitors of monoamine oxidase, like moclobemide and toloxatone, are deemed less interactive. Nonetheless, the risk of SS upon combination with other serotonin agents is tripled [11]. SS resulting from a therapeutic dose of one drug in solo is considered an idiosyncratic reaction due to individual vulnerability, while the most common SS occurs as a result of drug intoxication due to intentional self-poisoning or un-intentional drug–drug interactions in chronic patients treated with a serotoninergic agent [33]. Physicians should be vigilant and advise the patient on MAOIs to revisit the physician before taking any over-the-counter cough medications like the antitussive dextromethorphan, where some cases of SS have been reported [14]. Additionally, clinicians should allow four weeks spacing before starting serotonergic agents after MAOI cessation [19].

Aside from MAOIs, the association between SS and other antidepressants cannot be ignored. It can be highly presumed that antidepressants are the primary drug associated with SS. Aside from amitriptyline, most TCAs induce serotonergic effects, particularly when combined with MAOIs. The former had a 1000 affinity for the human cloned serotonin transporter [32]. Although SSRIs were privileged for their safety compared to the old-generation antidepressants [15], a large number of SSs occurred after exposure to a single SSRI or a combination of SSRI and other teratogenic agents like MAOIs and opioids [16,17,33,34,35]. Citalopram, fluoxetine, sertraline, escitalopram, and paroxetine are some examples of SSRIs associated with SS [10]. Tetracyclic antidepressants like mirtazapine were deemed safer and did not precipitate SS in an earlier study [14].

In agreement with the current study in which SS resulted from combining tramadol and SSRI, SS was described in three cases after exposure to this combination [36]. Schifano et al. reported that SS may result from some types of opioids, particularly when combined with antidepressants. Tramadol, a synthetic piperidine opioid, is a pro-serotonergic agent that has a weak serotonin re-uptake inhibitory effect, in addition to its ability to augment the release of serotonin through inhibition of gamma amino butyric acidergic inhibitory effects on serotonin neurons [33].

Of note, the current study reported that SS was significantly associated with acute amphetamine and MDMA poisoning. Among the recreational drugs, MDMA derivatives were considered the most common drug of abuse associated with SS, owing to their activation of the serotoninergic pathway, potentiating the activity of serotonin, dopamine, and norepinephrine [35]. Aside from tramadol, amphetamine, and MDMA, the association between SS and other illicit drugs like cocaine, LSD, cathinone, and aminoindane abuse was mentioned in a previous study [9].

The ethical implications of drug misuse in the context of SS and acute drug poisoning are multifaceted and deserve careful consideration [37]. Although symptoms and signs of SS develop rapidly within 24 h, this presentation is typically unusual among patients suffering from SS related to illicit drug use. Delay in seeking medical advice is common due to the similarity between SS clinical features and a broad spectrum of normal drug reactions generally perceived by illicit drug users, especially psychostimulants and sympathomimetics [35]. Clinical toxicologists should be aware of this delay as the patient’s condition might rapidly deteriorate, raising a failure to uphold the ethical principle of non-maleficence.

Silins et al. established a hierarchy of risk to classify serotonergic substances according to the risk of SS in concomitant use with ecstasy. SSRIs, SNRIs, TCAs, opioid analgesics, and antihistamines were described as low-risk combinations. The illicit drug cocaine is of intermediate risk, while MAOIs were considered as co-ingestants of high risk. Thus, psychiatrists are advised to prioritize drugs with low or minimal risks. Additionally, the involved physicians should be vigilant in screening their patients for illicit drugs before prescribing antidepressant drugs and other serotonin modulators [37]. Nevertheless, using the lowest effective doses of serotonergic agents, avoiding drug combinations whenever possible, assessing drug monographs for tapering and wash-out periods, and following up with the patients after adding new agents are other valid recommendations to mitigate the risk of developing SS [38]. Furthermore, the healthcare settings should be equipped with a 5-HT antagonist. In case the diagnosis is ambiguous, physicians should discontinue any serotonergic agents and start supportive care [39].

Harm reduction approaches are not restricted to the physician but extend to the patients. These approaches offer an ethically sound framework for minimizing the negative consequences, such as SS, associated with illicit drug use rather than criminalizing drug abuse. Providing comprehensive orientation campaigns to improve understanding of the risks associated with illicit drug use, including SS, is advised [40].

The current study reported a median delay time from exposure until approaching the emergency treatment of about 6 h. Mason et al. reported that about 60% of patients with SS became symptomatic 6 h after exposure [41]. However, delayed onset after 72 h was reported [8]. Nonetheless, the current study failed to address a significant correlation between the amount of delay until emergency treatment and the severity of clinical presentation, indicated by the GCS and PSS.

Although we did not report the length of SS episodes, it was inferred in previous studies that it is linked to the duration of action and half-life of the consumed drugs. This can explain the significant prolonged length of hospital stay among patients with SS, where the co-ingestion was a significant finding [14]. Sternbach reported that SS revokes within 24 h after cessation of the causative agent and initiation of treatment, through the signs and symptoms might last longer in a few preparations with longer duration and drugs with active metabolites [19].

Consistent with the current study, an earlier study reported that a PSS of more than two was associated with poor outcomes among acute poisoning patients. The patients with higher PSS showed significantly lower GCS and HCO_3_ levels, which support the role of both predictors as poor outcome indicators [42]. Albeit the current study inferred that the PSS was a good predictor of SS, we could not ignore the criticism that this score received. PSS was designed to assess the severity of all types of drug poisoning, which have different actions. Some reported disadvantages are the subjective nature of the included criteria and the time-consuming nature. In addition, the PSS contains many data points from 12 body organs with significant rater variability [43].

We must recognize that one score could not be applied to predict all adverse outcomes in different poisons. Thus, Ponnusankar et al. advised using PSS as a basic model to generate more accurate predictive scores tailored for specific poisons [44]. Additionally, concerns were raised regarding the validity of the original PSS. Many investigators misapplied or altered the PSS from the standard scoring. The static nature of PSS is another disadvantage. This score uses the worst physiologic data points at a particular time. Considering the critical nature of acute poisoning and the effect of rapid intervention on reducing adverse outcomes make the utility of PSS questionable [43].

Studies designed to explore other predictors of adverse outcomes and compare them to PSS in acute toxic exposure are scarce [45,46]. An earlier study conducted by Sharif et al. was not the only one, but it was one of the recent studies that adopted a simpler modified PSS as an adverse outcome predictor [47]. An earlier study concluded that PSS was inferior to the APACHE II score in predicting severe clinical outcomes due to poisoning. Sundari and Adithyan recommended re-placing PSS with the APACHE II score in different poison centers due to its wide range of variables, including the patient’s underlying co-morbid conditions and organ dysfunction [48]. Familiarity is another factor contributing to using the APACHE II score in all patients compared to PSS, which is only applicable to poisoned patients [43]. Another study compared PSS performance with other screening systems and showed comparable performance [49].

However, the primary reason behind inventing PSS was not to use it as an outcome predictor [43]. Thus, we hypothesized a need to propose simpler, subjective predictors instead of using PSS as an outcome predictor, particularly in severe illnesses like SS.

The present work showed that GCS was a significant discriminator of SS. Serotonin has a pivotal role in memory, stress, addiction, and nociception. Excessive accumulation of serotonin is associated with agitation, disturbed consciousness, and hallucination [50]. The association between disturbed consciousness, indicated by low GCS and SS was consistent with previous studies [24,51,52]. Although mental affection is one of the primary criteria for diagnosing SS [27], dependance on mental criteria increases the chance of falsely positively diagnosed SS, partially in patients exposed to drugs with anticholinergic effects [11]. Despite criticizing the GCS for being non-specific for SS, its utilization, along with the other proposed criteria, may offer a tool for monitoring improvement and response to treatment [11].

The present study conveys that tachycardia was a significant predictor of SS, which agrees with many previous studies reporting an association between tachycardia and SS [10,12,28]. An earlier study hypothesized that a substantial amount of serotonin is produced peripherally in the heart [53]. Increased cardiac properties are attributed to the augmentation of physiological response due to the accumulation of serotonin on different 5-HT receptors. Serotonin induces positive inotropic and chronotropic effects on the heart, carrying a significant risk of tachyarrhythmia if accumulated. These effects are mediated through the activation of 5-HT_4_ in the heart, which stimulates the guanosine tri-phosphate proteins and activates the adenylyl cyclase, enhancing the formation of cAMP and activation of protein kinases. These events are followed by the phosphorylation of L-type calcium channels, allowing calcium influx into the cytosol and increasing the calcium storage in the cardiac myocytes. The calcium binds to myofilaments and enhances the force and rate of cardiac contractions [54]. In the sinoatrial node, the activation of 5-HT_4_ receptors also leads to the activation of hyperpolarization-activated cyclic nucleotide-gated channels [55]. More precisely, the serotonin augments the funny currents in the atrial cells, allowing Na+ and K+ to leak into the sinoatrial cells, AV-nodal cells, and Purkinje fibers. These currents are known as the pacemaker currents, and upon activation of 5-HT_4_ receptors and cAMP formation, the current–voltage curve is shifted to more negative potentials [56,57].

Another reported pathophysiological mechanism explaining SS associated tachycardia is attributed to the autonomic derangement-like state associated with SS [58]. This tachycardia is part of the involved physiological hyperadrenergic response, as confirmed by dramatic amelioration after infusing beta-blockers such as esmolol and sedatives such as diazepam. Long-acting beta blockers are undesirable as they mask tachycardia and prevent following up the patient’s condition [39]. Although tachycardia is a significant discriminator of SS, we recommend combining it with other significant laboratory predictors. Tachycardia, hypertension mydriasis, and other noticed clinical findings have been commonly seen in other poisoning conditions, including anticholinergics and sympathomimetics, which increase the chance of misdiagnosing SS in these patients [11].

It can be hypothesized that adopting one or more objective criteria (PCO_2_ and HCO_3_), which are part of routine laboratory investigations, seems more straightforward and less complicated than PSS and provides a comparable prediction of SS even if the mental status is altered. Sun-Edelstein considered declaring PCO_2_ and metabolic acidosis, indicated by declining HCO_3_, as bad omen signs indicating life-threatening SS [14]. In alignment with the obtained findings, a severe decline in HCO_3_ level (base excess of −24.8, and HCO_3_ = 6.5) was reported in a female diagnosed with SS following exposure to multiple drugs [24]. Mikkelsen et al. described the low bicarbonate level as a nonspecific laboratory marker associated with SS [10].

Metabolic acidosis indicated by a decline in bicarbonate is an association described with SS resulting from methadone combinations with loxapine and lorazepam [59], tetrahydrocannabinol (THC) [60], and combination of alcohol and THC [61]. The mechanism of metabolic acidosis associated with SS is unknown; it was described as a non-specific common association [62]. Nonetheless, several mechanisms have been described in the literature. The muscular rigidity, tremors, and hyperreflexia characterizing SS lead to increased production of lactic acid, a byproduct of anaerobic metabolism, which can accumulate in the blood and contribute to metabolic acidosis. This temporal association is supported by the frequent association between rhabdomyolysis and seizure and metabolic acidosis in severe cases of SS [62]. Hyperthermia is another indirect cause of metabolic acidosis and declining bicarbonate levels. Hyperthermia increases metabolic demands and enhances energy production by up-regulation, leading to hypoxia at the cellular level, resulting in anaerobic respiration and subsequent metabolic acidosis. Hyperthermia in SS is intensely related to alteration of the mitochondrial energy handling [63].

Consistent with the current study, Koekkoek and Tajan reported a case of SS admitted following exposure to an overdose of drug combinations, including sertraline, and presented with low PCO_2_ level [24]. As a neurotransmitter, serotonin modulates the central respiratory drive by controlling the function of rhythm-generating respiratory neurons in the brainstem. This effect is achieved through activating 5-HT_4_ receptors. In addition, serotonin enhances pulmonary vascular resistance and remodels the tone of pulmonary vasculature. This modulation may occur directly by forming covalent bonds with intracellular signaling proteins in pulmonary vascular smooth muscle cells or secondary to hypoxia [50]. There is evidence that serotonin neurons are chemoreceptors that detect minute changes in blood CO_2_. Stimulation of these neurons induces an excitatory effect on breathing and provides a tonic drive to maintain respiration [64].

In critical care settings, SS induces a state mimicking the autonomic derangements in the form of irregular breathing patterns in which the rate and depth of respiration is disturbed [58]. The noticed low level of PCO_2_ among patients with SS is attributed to hyperventilation. Several case studies reported that hyperventilation was a significant finding preceding the diagnosis of SS. Hyperventilation is due to autonomic dysfunction and altered mental status associated with SS [34,60].

Increasing the rate and depth of respiration allows more CO₂ exhaling than is being produced by the body. This leads to a decrease in arterial PCO₂, resulting in hypocapnia. 5-HT neurons in the medulla and midbrain increase the firing rate in response to acidosis [65]. The noticed decline in PCO_2_ might be a compensatory mechanism in the trial to increase the pH if the patient experiences metabolic acidosis. This is supported by studies on animal models and human research, which revealed that medullary 5-HT neurons are chemoreceptors sensitive to changes in pH [66]. Stimulation of central serotonin in the brain stem triggers adaptive responses through their projections into respiratory nuclei, thereby driving respiratory adaptations [67].

Contradicting the obtained findings, Chrétien et al. described the hypercapnia in a case of SS [59]. Additionally, it was reported that rising PCO_2_ levels in patients with SS is an emergency condition that warrants an impending respiratory failure [11]. These findings may be explained on the basis of experimental studies showing that disruption of SS blunts the hypercapnic ventilatory response [66]. Understanding the complex nature of 5-HT receptors and their role in modulating the respiratory and metabolic hemostasis, which is disturbed due to the excessive neuronal firing in SS, explains the discrepancy in CO_2_ changes along with SS that was found in previous studies.

The current study elaborated that all ventilated patients suffered from SS. Respiratory failure in those patients may be attributed to associated truncal rigidity, affecting respiratory mechanisms and necessitating elective neuromuscular paralysis through mechanical ventilation [11]. Shock is another common associated sequela [14]. One reason for ICU admission in patients with life-threatening SS is to induce neuromuscular paralysis and orotracheal intubation. Neuromuscular paralysis is essential for assisting artificial respiration and as a treatment tool for associated hyperthermia, which initially results from excessive muscular contraction rather than changes in the hypothalamic heart regulating center [14].

Eventually, although being a less accurate predictor, the current study showed that SS was associated with a significantly higher RBC count, and RBCs could be a significant predictor for SS. Similarly, an earlier study reported an increased hematocrit level in a patient with SS [24]. It is well known that hematocrit measures the volume of red blood cells relative to whole blood and is used to identify conditions like polycythemia. Hence, the reported increased hematocrit supports the increased RBC count [68]. Neumann mentioned that serotonin is not only present in platelets, but also all cell constituents of blood contain some serotonin [54].

The primary cause of increased RBC count in SS may be due to the positive effects of serotonin on erythropoiesis. Serotonin potentiates the production and release of erythropoietin hormone that regulates RBC growth and maturation in the bone marrow [69]. We can hypothesize that increased erythropoiesis may be a secondary compensatory mechanism to increase oxygen delivery to the tissues to overcome SS-associated hypoxia. Experimental works showed that 5-HT is an effective antioxidant that increases RBC survival through a non-receptor-mediated mechanism. This explanation is supported by the reduced life span and lowered count of RBCs associated with the reduction of 5-HT circulating levels. The shortened survival was attributed to a decrease in the plasma antioxidant capacity due to decreased 5-HT [70]. The increased serotonin level in SS and increased RBC count could be correlated, given that most of the used drugs aim to increase the levels of serotonin centrally and peripherally.

## 5. Conclusions

Although SS is a potentially life-threatening condition, it is also a preventable one. Early discrimination of patients at risk is crucial. SS is frequent among older patients, suicide attempters, and those who try to obtain inebriation. SS was characterized by severe presentation (lower GCS, higher grades PSS) and sympathetic hyperactivity in the form of tachycardia, hypertension, hyperthermia, and tachypnea. These clinical features were typically seen in patients diagnosed with SS following exposure to single or multiple agents. Moreover, PSS was a significant predictor of SS. In addition, low PCO_2_, high pulse, low GCS and HCO_3_, and high RBCs were other significant predictors of SS. Combinations of serotonergic agents increase the likelihood of developing SS. Acute exposure to MAOIs, MDMA, TCA, tramadol, and dextromethorphan is a risk compared to SNRIs, and patients on long-term therapy with MAOIs, amphetamines, TCAs, and SSRIs are more vulnerable to SS. Clinicians should be vigilant when prescribing a combination of serotonergic therapy. The risk of SS increases in patients on illicit drugs such as MDMA, amphetamine, and cannabinoids or those using antitussive and over-the-counter medications such as dextromethorphan.

## 6. Limitations and Recommendations

The current study is a single-center study carried out on a limited number of patients. However, the retrospective design at one center may raise the bias of regional specificity. Therefore, we recommend a replication from multicenter prospective studies with double-blinded protocols, enrolling more diverse population. Despite these few imitations, the obtained findings may catalyze further studies to explore the various aspects of SS and promote the development of standardized diagnostic criteria for SS.

## Figures and Tables

**Table 1:** Predicted serotonin syndrome using Hunter Serotonin Toxicity Criteria versus serotonin syndrome as determined by a clinical toxicologist and by the need for 5-HT_2A_ antagonist.

		Diagnosis of Serotonin Syndrome Based on the Hunter Serotonin Toxicity Criteria			
		No	Yes	Total	Accuracy
	Actual	91	21	112	
Diagnosis of serotonin syndrome by a clinical toxicologist, as per medical records	No	91	5	96	Sensitivity = 76.2%
	Yes	0	16	16	Specificity = 100%
	Total	91	21	112	
Diagnosis of serotonin toxicity according to the treatment with a 5-HT_2A_ antagonist	No	83	9	92	Sensitivity = 57.1%
	Yes	8	12	20	Specificity = 91.2%
	Total	91	21	112	

**Table 2:** Demographics, history of toxic exposure, vital signs, and scoring of the studied patients on admission.

	Total (n = 112)		No serotonin Syndrome (n = 91)		Serotonin Syndrome (n = 21)		Test of sig.	*p*
Age (years)							U 472.5	<0.001 *
Mean ± SD.	29.2 ± 12.47		27.3 ± 12.13		37.1 ± 10.91			
Min.–Max.	18.0–76.0		18.0–76.0		18.0–54.0			
Median (IQR)	25.0 (19.0–35.75)		24.0 (18.0–32.0)		39.0 (29.5–46.0)			
Sex	No.	%	No.	%	No.	%	χ^2^ 2.999	0.083
Female	51	45.5	45	49.5	6	28.6		
Male	61	54.5	46	50.5	15	71.4		
Manner of exposure							MC	0.004 *
Unintentional	23	20.5	20	22.0	3	14.3		
Suicidal	46	41.1	35	38.5	11	52.4		
To obtain inebriation	12	10.7	6	6.6	6	28.6		
Undetermined	31	27.7	30	33.0	1	4.8		
Long-term co-ingestion	49	43.8	35	38.5	14	66.7	χ^2^ 6.476	0.011 *
Delay time between exposure and receiving treatment (hr.)							U 858.5	0.461
Mean ± SD.	9.4 ± 15.53		9.7 ± 15.77		8.3 ± 14.74			
Min.–Max.	0.5–120.0		0.5–120.0		3.0–72.0			
Median (IQR)	6.0 (4.0–6.75)		6.0 (4.0–7.0)		5.0 (4.0–6.0)			
Systolic blood pressure							t 2.028	0.045 *
Mean ± SD.	117.6 ± 18.38		115.9 ± 18.85		124.8 ± 14.44			
Min.–Max.	80.0–176.0		80.0–176.0		100.0–150.0			
Median (IQR)	116.0 (105.0–129.75)		115.0 (102.0–125.0)		126.0 (110.5–138.0)			
Diastolic blood pressure							t 2.301	0.023 *
Mean ± SD.	74.3 ± 13.71		72.8 ± 13.88		80.4 ± 11.31			
Min.–Max.	42.0–102.0		42.0–102.0		65.0–102.0			
Median (IQR)	75.0 (66.0–82.75)		75.0 (60.0–80.0)		83.0 (70.0–90.0)			
Axillary body Temperature °C							t 12.221	<0.001 *
Mean ± SD.	37.3 ± 1.07		36.9 ± 0.68		39.0 ± 0.80			
Min.–Max.	34.5–40.1		34.5–40.0		36.6–40.1			
Median (IQR)	37.0 (36.7–37.7)		36.9 (36.7–37.1)		39.0 (38.8–39.5)			
O2 saturation							t 1.784	0.077
Mean ± SD.	97.8 ± 2.43		97.9 ± 2.45		96.9 ± 2.21			
Min.–Max.	88.0–100.0		88.0–100.0		90.0–100.0			
Median (IQR)	98.0 (97.0–99.0)		98.0 (97.0–100.0)		97.0 (95.5–98.0)			
Respiratory rate (cycle/min)							t 4.154	<0.001 *
Mean ± SD.	21.0 ± 3.90		20.3 ± 3.50		24.0 ± 4.21			
Min.–Max.	12.0–37.0		12.0–30.0		18.0–37.0			
Median (IQR)	20.0 (18.0–24.0)		20.0 (18.0–23.0)		24.0 (21.0–26.5)			
Pulse (beat/minute)							t 4.610	<0.001 *
Mean ± SD.	101.2 ± 19.04		97.5 ± 17.89		117.1 ± 15.71			
Min.–Max.	57.0–151.0		57.0–140.0		99.0–151.0			
Median (IQR)	100.0 (90.0–115.0)		95.0 (86.0–109.0)		118.0 (100.0–129.0)			
GCS							U 408.0	<0.001 *
Min.–Max.	3.0–15.0		3.0–15.0		8.0–15.0			
Median (IQR)	15.0 (13.0–15.0)		15.0 (13.0–15.0)		13.0 (9.0–13.0)			
PSS	No.	%	No.	%	No.	%	χ^2^ 51.091	<0.001 *
Minor	53	47.3	52	57.1	1	4.8		
Moderate	43	38.4	36	39.6	7	33.3		
Severe	16	14.3	3	3.3	13	61.9		

χ^2^: Chi square test; FE: Fischer exact test; IQR: interquartile range; MC: Monte Carlo exact test; * *p* < 0.05 (statistically significant); U: Mann–Whitney U test.

**Table 3:** The presenting complaints, and clinical findings of the studied patients on admission.

	Total (n = 112)		No Serotonin Syndrome (n = 91)		Serotonin Syndrome (n = 21)		Test of sig.	*p*
Gastrointestinal symptoms	No.	%	No.	%	No.	%	χ^2^ 17.699	<0.001 *
Vomiting, colic, diarrhea, constipation	55	49.1	36	39.6	19	90.5		
Respiratory affection							MC	0.392
Chest tightness and breathlessness	9	8.0	6	6.6	3	14.3		
Cough	2	1.8	2	2.2	0	0.0		
Cardiovascular manifestations							MC	0.010 *
Chest pain	5	4.5	4	4.4	1	4.8		
Palpitation	21	18.8	12	13.2	9	42.9		
Central nervous system manifestations							MC	<0.001 *
Decreased consciousness level	43	38.4	27	29.7	16	76.2		
Lost consciousness	17	15.2	13	14.3	4	19.0		
Seizures	27	24.1	16	17.6	11	52.4	χ^2^ 11.293	<0.001 *
Extrapyramidal manifestations							χ^2^ 26.266	<0.001 *
Rolling of eye, clenched jaw, dystonia, neck spasm, etc.	30	26.8	15	16.5	15	71.4		
Clonus							MC	<0.001 *
Inducible	12	10.7	4	4.4	8	38.1		
Spontaneous	3	2.7	0	0.0	3	14.3		
Diaphoresis	24	21.4	12	13.2	12	57.1	FE	<0.001 *
Agitation	23	20.5	12	13.2	11	52.4	FE	<0.001 *
Shivering	34	30.4	18	19.8	16	76.2	χ^2^ 25.682	<0.001 *
Ocular clonus	17	15.2	5	5.5	12	57.1	FE	<0.001 *
Tremors	16	14.3	8	8.8	8	38.1	FE	0.002 *
Hyperreflexia	5	4.5	0	0.0	5	23.8	FE	<0.001 *
Hypertonicity	8	7.1	0	0.0	8	38.1	FE	<0.001 *
Pupil							χ^2^ 59.134	<0.001 *
Normal	75	67.0	73	80.2	2	9.5		
Miosis	9	8.0	9	9.9	0	0.0		
Mydriasis	28	25.0	9	9.9	19	90.5		
ECG findings							χ^2^ 33.924	<0.001 *
Normal sinus rhythm	68	60.7	67	73.6	1	4.8		
One or more forms of arrhythmias	44	39.3	24	26.4	20	95.2		

χ^2^: Chi square test; FE: Fischer exact test; MC: Monte Carlo exact test; * *p* < 0.05 (statistically significant); U: Mann–Whitney U test.

**Table 4:** Laboratory investigations of the studied patients on admission.

	Total (n = 112)	No Serotonin Syndrome (n = 91)	Serotonin Syndrome (n = 21)	Test of sig.	*p*
Random blood glucose level (mmol/L)				t 1.459	0.147
Mean ± SD.	4.9 ± 1.09	4.9 ± 1.06	4.6 ± 1.20		
Min.–Max.	2.3–8.9	2.3–8.9	2.9–6.7		
Median (IQR)	4.8 (4.2–5.6)	4.9 (4.3–5.8)	4.4 (3.64–5.6)		
Na (mmol\L)				t 0.081	0.936
Mean ± SD.	137.1 ± 3.53	137.1 ± 3.45	137.2 ± 3.95		
Min.–Max.	121.0–145.0	121.0–145.0	128.0–145.0		
Median (IQR)	138.0 (136.0–139.0)	138.0 (135.0–139.0)	138.0 (136.0–139.0)		
K (mmol\L)				t 0.119	0.905
Mean ± SD.	3.9 ± 0.43	3.9 ± 0.42	3.9 ± 0.47		
Min.–Max.	2.8–5.5	3.0–5.5	2.8–4.68		
Median (IQR)	3.9 (3.7–4.2)	3.9 (3.7–4.2)	4.09 (3.76–4.3)		
Total bilirubin level (umol/L)				U 789.5	0.216
Mean ± SD.	10.1 ± 5.61	9.9 ± 5.73	10.9 ± 5.07		
Min.–Max.	2.6–25.9	2.6–25.9	3.8–22.0		
Median (IQR)	8.9 (5.8–13.0)	8.4 (5.7–12.0)	10.5 (6.55–14.55)		
SGOT (U/L)				U 952.5	0.982
Mean ± SD.	66.1 ± 394.42	74.4 ± 437.56	29.9 ± 11.84		
Min.–Max.	10.0–4202.0	10.0–4202.0	15.0–58.0		
Median (IQR)	29.0 (22.0–33.0)	29.0 (22.0–33.0)	30.0 (19.5–33.5)		
SGPT (U/L)				U 675.5	0.036 *
Mean ± SD.	55.4 ± 366.60	62.6 ± 406.76	24.4 ± 10.26		
Min.–Max.	7.0–3899.0	7.0–3899.0	7.0–43.0		
Median (IQR)	19.0 (16.0–22.0)	19.0 (15.0–22.0)	22.0 (18.0–34.0)		
Serum urea (mmol/L)				U 921.5	0.800
Mean ± SD.	4.1 ± 1.83	4.0 ± 1.67	4.2 ± 2.45		
Min.–Max.	1.1–13.2	1.1–10.0	1.8–13.2		
Median (IQR)	3.6 (2.9–5.0)	3.6 (2.9–5.0)	3.4 (2.65–5.05)		
Serum creatinine (umol/L)				U 917.0	0.773
Mean ± SD.	66.9 ± 24.24	66.3 ± 24.08	69.3 ± 25.40		
Min.–Max.	40.0–181.0	40.0–181.0	42.0–135.0		
Median (IQR)	59.0 (49.0–78.0)	58.0 (49.0–78.0)	62.0 (49.0–88.5)		
pH				0.407	0.684
Mean ± SD.	7.4 ± 0.08	7.4 ± 0.08	7.4 ± 0.08		
Min.–Max.	6.9–7.503	6.9–7.49	7.2–7.503		
Median (IQR)	7.37 (7.35–7.41)	7.37 (7.35–7.41)	7.36 (7.31–7.4)		
HCO_3_ (mEq/L)				3.816	<0.001 *
Mean ± SD.	22.2 ± 2.92	22.6 ± 2.81	20.1 ± 2.53		
Min.–Max.	14.0–32.0	14.0–32.0	16.0–24.2		
Median (IQR)	22.85 (20.0–24.0)	23.1 (20.5–24.2)	19.0 (18.5–22.5)		
PCO_2_ (mmHg)				4.567	<0.001 *
Mean ± SD.	40.0 ± 8.51	41.7 ± 7.93	33.0 ± 7.40		
Min.–Max.	20.0–62.0	23.0–62.0	20.0–51.0		
Median (IQR)	39.0 (35.0–45.0)	40.0 (36.0–45.0)	31.0 (29.0–34.85)		
Hemoglobin (gram/dL)				t 1.433	0.155
Mean ± SD.	13.5 ± 1.61	13.4 ± 1.61	13.9 ± 1.56		
Min.–Max.	9.4–17.7	9.4–17.7	10.72–16.8		
Median (IQR)	13.2 (12.6–14.6)	13.2 (12.5–14.5)	13.8 (12.7–15.2)		
RBCs (million/microliter)				t 3.234	0.002 *
Mean ± SD.	4.8 ± 0.61	4.7 ± 0.50	5.1 ± 0.89		
Min.–Max.	3.82–8.32	3.82–6.48	4.29–8.32		
Median (IQR)	4.63 (4.35–4.95)	4.54 (4.34–4.90)	4.9 (4.60–5.33)		
WBCs * 1000/microliter				U 747.0	0.120
Mean ± SD.	9.6 ± 3.91	9.3 ± 3.57	11.2 ± 4.94		
Min.–Max.	3.73–21.17	3.73–19.4	5.26–21.17		
Median (IQR)	8.26 (6.80–11.83)	8.05 (6.52–11.8)	9.81 (7.08–15.85)		
Platelet count * 1000/microliter				U 944.5	0.935
Mean ± SD.	305.0 ± 86.94	306.7 ± 89.67	297.9 ± 75.51		
Min.–Max.	152.0–622.0	152.0–622.0	152.0–415.0		
Median (IQR)	299.5 (239.25–364.0)	297.0 (240.0–364.0)	305.0 (227.0–371.0)		

U: Mann–Whitney U test; IQR: interquartile range; t: independent *t* test; * *p* < 0.05 (statistically significant).

**Table 5:** Therapeutic measures and length of hospital stay among the studied patients.

	Total (n = 112)		No Serotonin Syndrome (n = 91)		Serotonin Syndrome (n = 21)		Test of sig.	*p*
	No.	%	No.	%	No.	%		
Treatment with 5-HT_2A_ antagonist	20	17.9	8	8.8	12	57.1	FE	<0.001 *
Need for mechanical ventilation	7	6.3	0	0.0	7	33.3	FE	<0.001 *
ICU admission	31	27.7	10	11.0	21	100.0	χ^2^ 67.533	<0.001 *
Length of hospital stay from admission until discharge							U 281.0	<0.001 *
Mean ± SD.	26.0 ± 34.73		18.0 ± 14.56		60.6 ± 64.77			
Min.–Max.	4.0–308.0		4.0–72.0		6.0–308.0			
Median (IQR)	17.0 (8.0–29.75)		13.0 (7.0–24.0)		48.0 (24.0–72.0)			

χ^2^: Chi square test; FE: Fischer exact test; U: Mann–Whitney U test; * *p* < 0.05 (statistically significant).

**Table 6:** The vital signs, presenting complaints, and clinical findings of the patients diagnosed with serotonin syndrome on admission.

	Serotonin Syndrome (Single Administration) (n = 7)		Serotonin Syndrome (Drug Combination) (n = 14)		Test of sig.	*p*
Systolic blood pressure					t 1.437	0.167
Mean ± SD.	118.6 ± 13.25		127.9 ± 14.43			
Min.–Max.	100.0–141.0		103.0–150.0			
Median (IQR)	119.0 (110.0–126.0)		130.0 (117.5–139.25)			
Diastolic blood pressure					t 0.967	0.346
Mean ± SD.	77.0 ± 13.88		82.1 ± 9.93			
Min.–Max.	65.0–102.0		67.0–95.0			
Median (IQR)	71.0 (67.0–90.0)		86.0 (70.75–90.0)			
Axillary body Temperature C0					t 1.002	0.329
Mean ± SD.	39.3 ± 0.52		38.9 ± 0.90			
Min.–Max.	38.8–40.0		36.6–40.1			
Median (IQR)	39.1 (38.8–40.0)		39.0 (38.57–39.32)			
O2 saturation					t 1.199	0.245
Mean ± SD.	97.7 ± 1.60		96.5 ± 2.41			
Min.–Max.	95.0–100.0		90.0–99.0			
Median (IQR)	98.0 (97.0–99.0)		97.0 (95.0–98.0)			
Respiratory rate (cycle/min)					t 0.215	0.832
Mean ± SD.	24.3 ± 2.69		23.9 ± 4.88			
Min.–Max.	20.0–28.0		18.0–37.0			
Median (IQR)	24.0 (22.0–26.0)		22.5 (20.0–27.0)			
Pulse (beat/minute)					t 2.093	0.052
Mean ± SD.	126.4 ± 17.35		112.4 ± 13.02			
Min.–Max.	99.0–151.0		100.0–135.0			
Median (IQR)	122.0 (120.0–145.0)		110.0 (100.0–127.0)			
GCS					U 38.0	0.392
Min.–Max.	9.0–13.0		8.0–15.0			
Median (IQR)	12.0 (9.0–13.0)		13.0 (9.0–13.25)			
PSS	No.	%	No.	%	MC	1.000
Minor	0	0.0	1	7.1		
Moderate	2	28.6	5	35.7		
Severe	5	71.4	8	57.1		
Gastrointestinal symptoms	No.	%	No.	%	FE	0.533
Vomiting, colic, diarrhea, constipation	7	100.0	12	85.7		
Respiratory affection					FE	1.000
Chest tightness and breathlessness	1	14.3	2	14.3		
Cough	0	0.0	0	0.0		
Cardiovascular manifestations					MC	0.028 *
Chest pain	1	14.3	0	0.0		
Palpitation	5	71.4	4	28.6		
Central nervous system manifestations					MC	0.152
Decreased consciousness level	4	57.1	12	85.7		
Lost consciousness	3	42.9	1	7.1		
Seizures	3	42.9	8	57.1	FE	0.659
Extrapyramidal manifestations					FE	1.000
Rolling of eye, clenched jaw, dystonia, neck spasm, etc.	5	71.4	10	71.4		
Clonus					MC	0.574
Inducible	2	28.6	6	42.9		
Spontaneous	2	28.6	1	7.1		
Diaphoresis	5	71.4	7	50.0	FE	0.642
Agitation	4	57.1	7	50.0	FE	1.000
Shivering	5	71.4	11	78.6	FE	1.000
Ocular clonus	5	71.4	7	50.0	FE	0.642
Tremors	1	14.3	7	50.0	FE	0.174
Hyperreflexia	2	28.6	3	21.4	FE	1.000
Hypertonicity	5	71.4	3	21.4	FE	0.056
Pupil					FE	0.533
Normal	0	0.0	2	14.3		
Mydriasis	7	100.0	12	85.7		
ECG findings					FE	1.000
Normal sinus rhythm	0	0.0	1	7.1		
One or more forms of arrhythmias	7	100.0	13	92.9		

χ^2^: Chi square test; FE: Fischer exact test; MC: Monte Carlo exact test; * *p* < 0.05 (statistically significant); U: Mann–Whitney U test.

**Table 7:** Univariate and multivariate regression analysis to identify predictors of serotonin syndrome.

Parameters	Univariate		Multivariate	
	B (95% CI)	*p*	B (95% CI)	*p*
Age (years)	0.010 (0.004–0.015)	<0.001 *	0.004 (0.0–0.009)	0.069
Manner of exposure	−0.034 (−0.1–0.033)	0.320		
Systolic blood pressure	0.004 (0.00009–0.008)	0.045 *	−0.002 (−0.006–0.002)	0.372
Diastolic blood pressure	0.006 (0.001–0.011)	0.023 *	0.002 (−0.003–0.008)	0.392
Respiratory rate (cycle/min)	0.037 (0.019–0.055)	<0.001 *	0.008 (−0.009–0.026)	0.329
Pulse (beat/minute)	0.008 (0.005–0.012)	<0.001 *	0.005 (0.001–0.008)	0.009 *
GCS	−0.037 (−0.062–−0.011)	0.005 *	−0.037 (−0.058–−0.016)	<0.001 *
SGPT (ALT) (U/L)	0.0 (0.0–0.0)	0.669		
HCO_3_ (mEq/L)	−0.046 (−0.07–−0.022)	<0.001 *	−0.033 (−0.053–−0.013)	0.001 *
PCO_2_ (mmHg)	−0.018 (−0.026–−0.01)	<0.001 *	−0.012 (−0.019–−0.004)	0.003 *
RBC count (million/microliter)	0.188 (0.073–0.304)	0.002 *	0.145 (0.051–0.239)	0.003 *
PSS	0.334 (0.251–0.416)	<0.001 *		

* *p* < 0.05 (Statistically significant).

**Table 8:** Receiver operating characteristic (ROC) curve analysis and pairwise comparison of AUC for predictors of serotonin syndrome.

	AUC	*p*	Cut Off	Sensitivity	Specificity	PPV	NPV	Accuracy
PSS	0.879	<0.001 *	>2.5	61.9%	96.7%	81.3%	91.7%	90.2%
PCO_2_ (mmHg)	0.816	<0.001 *	<35.85	78.0%	81.0%	94.7%	45.9%	78.6%
Pulse (beat/minute)	0.796	<0.001 *	>109.5	66.7%	75.8%	38.9%	90.8%	74.1%
GCS	0.786	<0.001 *	<14.0	73.6%	85.7%	95.7%	42.9%	75.8%
HCO_3_ (mEq/L)	0.758	<0.001 *	<21.5	69.2%	71.4%	91.3%	34.9%	69.6%
RBCs (million/microliter)	0.708	0.003 *	>4.705	71.4%	65.9%	32.6%	90.9%	66.9%
Pair wise comparison of AUC								
	PSS	PCO_2_	Pulse (beat/minute)	GCS	HCO_3_		RBCs (million/microliter)	
PSS	-	0.404	0.182	0.159	0.087		0.016 *	
PCO_2_ (mmHg)	0.404	-	0.794	0.706	0.488		0.196	
Pulse (beat/minute)	0.182	0.794	-	0.894	0.597		0.221	
GCS	0.159	0.706	0.894	-	0.709		0.299	
HCO_3_ (mEq/L)	0.087	0.488	0.597	0.709	-		0.528	
RBCs (million/microliter)	0.016 *	0.196	0.221	0.299	0.528		-	

AUC: Area under a curve; * *p* < 0.05 (statistically significant); NPV: Negative predictive value; PPV: Positive predictive value.

**Table 9:** Sensitivity analysis using an alternative statistical model (binary logistic regression).

Potential Predictors	Wald	Sig.	Odds Ratio	95% C.I for Odds Ratio	
				Lower	Upper
PSS	23.460	<0.001 *	17.380	5.474	55.189
PCO_2_ (mmHg)	7.650	0.006 *	0.847	0.754	0.953
Pulse (beat/minute)	7.245	0.007 *	1.063	1.017	1.111
GCS	9.848	0.002 *	0.680	0.534	0.865
HCO_3_ (mEq/L)	5.351	0.021 *	0.739	0.572	0.955
RBCs (million/microliter)	7.022	0.008 *	3.806	1.416	10.230

* *p* < 0.05 (Statistically significant).

## Data Availability

The raw data supporting the conclusions of this article will be made available by the authors on request. The views expressed in this paper are those of the authors and not do not necessarily reflect those of the SFDA or its stakeholders. Guaranteeing the accuracy and the validity of the data is a sole responsibility of the research team.
